# Association of Higher Circulating Insulin Antibody with Increased Mean Amplitude Glycemic Excursion in Patients with Type 2 Diabetes Mellitus: A Cross-Sectional, Retrospective Case-Control Study

**DOI:** 10.1155/2019/7304140

**Published:** 2019-03-18

**Authors:** Jian Zhu, Lu Yuan, Wen-ji Ni, Yong Luo, Jian-hua Ma

**Affiliations:** Department of Endocrinology, Nanjing First Hospital, Nanjing Medical University, Nanjing 210012, China

## Abstract

Insulin antibody (IA) may potentially affect a patient's glycemic control due to its variability in both binding and/or releasing insulin. However, the association between IA titer and daily glycemic variability (GV) is still unknown. We thus performed this cross-sectional, retrospective case-control study to assess the relationship between IA titer and mean amplitude glycemic excursion (MAGE) in type 2 diabetes mellitus (T2DM) patients using a continuous glucose monitoring (CGM) system. We recruited 100 eligible patients (IA > 5%, IA positive) and divided them into two groups—a low (L) group and a high (H) group—based on their IA titer. The control (C) group consisted of 47 patients (IA ≤ 5%, IA negative) matched for age, BMI, gender, and glycosylated hemoglobin A1c (HbA1c). The CGM determined the GV of enrolled patients. The primary outcome was the relationship between the IA titer and the MAGE, and the secondary outcome was the differences of GV among the three groups. We found that patients in the H group had higher levels of blood glucose fluctuation parameters than those in the L and C groups. The Ln(IA) was positively correlated with Ln(MAGE) even after adjusting for age, gender, BMI, HbA1c, and fasting and postprandial C-peptide(*r* = 0.423, *p* < 0.001). Multiple linear stepwise regression analysis revealed that Ln(IA) was an independent factor of Ln(MAGE) (beta = 0.405, *p* < 0.001). In conclusion, the higher circulating IA titer was associated with increased MAGE in T2DM patients, indicating that those patients with elevated IA titer should receive GV assessment and individualized treatment.

## 1. Introduction

Administration of exogenous animal insulin for the treatment of diabetes often induces the production of insulin antibodies (IA) [1, 2]. In recent years, the usage of recombinant human insulin preparations and human insulin analogues has significantly reduced but not entirely suppressed the incidence of IA development [3–6]. These antibodies might affect a patient's glycemic control due to their tendency to bind and/or release insulin in an unpredictable fashion [7–9]. Sporadic case reports [10–15] and some small-scale studies [16, 17] showed that individuals with high IA titer developed severe clinical consequences, such as extreme insulin resistance, hyperglycemia, and hypoglycemia episodes. Previous studies [3–6] on this topic have suggested that circulating IA rarely interfere with the glycemic control of patients, as most of them have low binding capacity and circulate at a relatively low titer. Nevertheless, it should be noted that these studies set HbA1c level and hypoglycemia episodes, not glycemic variability (GV), as their primary outcomes.

The ultimate goal of diabetes management is to reduce the risk of microvascular and macrovascular complications. Recent studies revealed that GV has more deleterious effects than sustained hyperglycemia in the pathogenesis of diabetic cardiovascular complications [18, 19]. Furthermore, a higher GV, which induces oxidative stress and endothelial dysfunction, is associated with increased incidence of diabetic microvascular complications at similar HbA1c levels [20–22]. Up to now, previous studies [23–26] demonstrated that insulin resistance, pancreatic islet beta cell function, and body mass index (BMI) act as independent predictors of GV. However, the association of IA titer with daily GV is not clear. Hence, we performed this single-center, retrospective case-control study to assess the relationship between IA titer and GV in T2DM patients through continuous glucose monitoring (CGM).

## 2. Methods

### 2.1. Study Design

This retrospective, cross-sectional case-control study was approved by the ethics committee of Nanjing First Hospital, Nanjing Medical University, which waived the requirement for written informed consent from the participants. All procedures followed were in accordance with the Declaration of Helsinki guidelines, including any relevant details. Two researchers extracted data from consecutive medical records of patients referred to our hospital. Data analysis covered the period from June 2016 to July 2018. Inclusion criteria for the IA-positive group (IA titer > 5%) included the following: (1) patient age is ≥18 years, (2) BMI was between 18 and 35 kg/m^2^, (3) insulin regimen was low premixed human insulin or insulin analogue (twice a day), (4) the history of usage of premixed human insulin or insulin analogue was longer than one year, (5) IA was negative before human insulin or insulin analogue treatment, (6) there are no changes in the type of insulin and oral antidiabetic drugs from 3 months before the end of index date, (7) oral antidiabetic drugs were metformin (0.5 g, thrice a day) and/or acrobose (50 mg, thrice a day), and (8) the patient had at least 24 h CGM data. Patients were excluded if they (1) were positive for antiglutamic acid decarboxylase antibodies; (2) had severe cardiovascular diseases, such as stroke and myocardial infarction, coronary artery bypass grafting, percutaneous coronary intervention, and heart failure; (3) had infectious diseases; (4) had acute complications of diabetes on admission, such as diabetic ketoacidosis and lactic acidosis; or (5) had severely impaired liver and kidney function and psychiatric disorders or were pregnant. Patients with maturity-onset diabetes in youth, mitochondrial diabetes mellitus, type 1 diabetes mellitus, cognitive disease, alcoholism, known cancers, and drug abuse issues were also excluded. The IA-negative control group consisted of subjects strictly matched for age, gender, BMI, and HbA1c.

### 2.2. Insulin Antibody Titer, C-peptide Level, and HbA1c Assessments

All serum samples for IA determination were collected from fasting individuals to minimize interference from insulin or insulin analogue administration. The IA titers were determined using the iodine-125 insulin antibody array kit (Beijing North Institute of Biological Technology, China) in accordance with the manufacturer's instructions, the procedure being similar to that in the previous studies [17, 27]. In brief, the serum sample (100 *μ*l) was diluted with buffer and then incubated with mono-^125^I (TyrA 14) human insulin (1.1 kbp, 100 *μ*l). After incubation at 37°C for 2 h, bound and free insulin fractions were separated by polyethylene glycol. The results were expressed in terms of bound radioactivity in the precipitate as a percentage of total counts in the assay. Blank values obtained by measurement of specific IA-zero serum were subtracted from sample values. IA titers of control sera always were below 5%. IA ≤ 5% was identified as a negative result, and IA > 5% as positive.

C-peptide levels were measured using Cobas e601 (Roche Diagnostics, Switzerland), and the lower limit of the reference value for fasting C-peptide level and 2 h-C-peptide level was 1.1 ng/ml. HbA1c levels were determined using high-performance liquid chromatography (Bio-Rad Company, Hercules, CA, USA).

### 2.3. Daily Glycemic Variability Parameters

CGM data was obtained with Medtronic Minimed CGM System Gold (Medtronic Incorporated, Northridge, USA), which was performed as described in a previous study [28]. We analyzed the data collected during the CGM covering the period from 7 a.m. day 2 to 7 a.m. day 3. The 24 h mean amplitude of glycemic excursion (MAGE) was calculated manually for each patient by measuring the arithmetic mean of the ascending and descending excursions between consecutive peaks and nadirs for the same 24 h period, and only absolute excursion values > 1 SD were considered, as previously described [28]. Other parameters, including 24 h mean blood glucose (MBG), standard deviation of 24 h blood glucose (SDBG), large amplitude of glycemic excursion (LAGE), glucose area under the curve above 13.9 mmol/l (AUC > 13.9) and below 3.9 mmol/l (AUC < 3.9), and the percentage of the time spent on glucose concentrations above 13.9 mmol/l (PT1) and below 3.9 mmol/l (PT2), were also calculated, respectively. The primary outcome was the relationship between the IA titer and the MAGE. The secondary endpoint was the difference in GV among the three groups.

### 2.4. Statistical Analysis

The sample size required was calculated using PASS 11.0. The level of significance, *α*, was set as 0.05, and the desired power of the study (1 − *β*) was 90%. Assuming that the mean of Ln(MAGE) was 1.4, 1.6, and 1.8 for the control, L, and H groups, respectively, the hypothesized standard deviation within a group was 0.50 and the minimum number of patients required was 123.

Data was analyzed with the SPSS PASW Statistics 22 Package. All continuous data were tested for normality using the Kolmogorov-Smirnov test. Normally distributed parameters were expressed as mean ± standard deviation, and nonnormally distributed parameters were expressed as median and range. Parameters that did not fulfill a normal distribution were mathematically transformed to improve symmetry for subsequent analyses. One-way ANOVA, nonparametric tests, and chi-square test had been used for difference analysis among groups, respectively. The relation between variables was analyzed by Pearson's correlation test. Multiple linear regression was made in a forward stepwise manner to select suitable variables in the model. Statistical analyses were two-sided, and *p* value < 0.05 was considered statistically significant.

## 3. Results

### 3.1. Baseline Characteristics of Patients

In total, one hundred and twelve IA-positive T2DM patients who met the inclusion criteria were recruited into the present study. Twelve patients were excluded because of inadequate CGM data. Finally, 100 patients (45 men and 55 women; age 62.90 ± 11.11 years, BMI 24.63 ± 3.22 kg/m^2^, and HbA1c values 8.49 ± 1.45%) were enrolled and subdivided into two groups according to IA titer as follows: a low (L) group with IA titers > 5% and ≤15.53% (50 patients) and a high (H) group with IA titers > 15.53% (50 patients). A total of 47, 50, and 50 subjects were allocated into the control (C), L, and H groups with IA titers of 2.97% (1.76%, 4.15%), 8.80% (6.48%, 11.76%), and 31.72% (21.80%, 43.72%), respectively. As shown in Table 1, there were no significant differences in age, gender, BMI, HbA1c level, duration of diabetes, fasting C-peptide (C-p 0′) level, 2 h postprandial C-p (C-p 120′) level, the number of patients with decreasing C-p 0′ level or decreasing C-p 120′ level, the ratio of human insulin over insulin analogues, and the ratio of oral antidiabetes drugs among the three groups.

### 3.2. Glycemic Variation Profiles

There were no statistically significant differences in MBG among the three groups. However, the MAGE (Ln(MAGE): 1.79 ± 0.50 (H) vs. 1.60 ± 0.35 (L), *p* = 0.032; 1.79 ± 0.50 (H) vs. 1.36 ± 0.37 (C), *p* < 0.001; and 1.60 ± 0.35 (L) vs. 1.36 ± 0.37 (C), *p* < 0.001, respectively) showed a significantly progressive increase alongside IA titer in T2DM patients with insulin therapy. Moreover, the H group had a significantly increased AUC > 13.9 and PT1 than the L group and C group. Similarly, the H group had a significantly increased SDBG, LAGE, AUC < 3.9, and PT2 than the C group. Although not statistically significant, there was a slightly increased tendency on SDBG, LAGE, AUC < 3.9, and PT2 in the H group as compared to the L group (Table 2).

### 3.3. Correlation and Regression Analysis

Pearson's correlation test showed that Ln(IA), BMI, HbA1c, Sqrt (C-p 0′), and Sqrt (C-p 120′) were correlated with the Ln(MAGE) in all subjects, respectively (Table 3); the Ln(IA) was still associated with Ln(MAGE) after adjustment for age, gender, BMI, HbA1c, Sqrt (C-p 0′), and Sqrt (C-p 120′) (*r* = 0.423, *p* < 0.001). We further treated Ln(MAGE) level as a dependent variable and then performed the multiple linear stepwise regression analysis to assess the independent effects of Ln(IA), age, gender, BMI, HbA1c, Sqrt (C-p 0′), and Sqrt (C-p 120′) on Ln(MAGE). Our data showed that Ln(IA) emerged as an independent variable associated with Ln(MAGE) and that Ln(IA) itself could predict 15.8% of Ln(MAGE) variance in T2DM patients (Table 4, model 1). As shown in model 3 (Table 4), Ln(IA), BMI, and HbA1c were the three independent predictors of Ln(MAGE).

## 4. Discussion

To the best of our knowledge, this was the first study to investigate the relationship between IA titer and MAGE in T2DM patients using a CGM system. The present study revealed a novel observation that, even at similar HbA1c and BMI levels, the MAGE gradually increased with IA titer in T2DM patients with low premixed insulin or insulin analogue therapy. We also observed that patients with IA titer above 15.53% exhibited more substantial blood glucose fluctuations. In addition, although there was no statistically significant difference on MBG and hypoglycemia episodes between low IA titer (>5% and ≤15.53%) patients and IA-negative subjects, the indexes of GV of the L group, including MAGE, SDBG, and LAGE, were significantly higher than those of the C group. Our data indicated that patients with higher IA titer should receive “precision medicine and individual therapy,” aimed at reducing the GV.

IA can be divided into two populations: low affinity/high capacity and high affinity/low capacity. The former is commonly found in patients with postprandial hyperglycemia and nocturnal hypoglycemia [13–15], whereas the latter seems likely to have less clinical significance in previous studies [3–6]. Likewise, there are two kinds of classical techniques used to measure IA in an everyday clinical setting: the enzyme-linked immunosorbent assay (ELISA) and the radioimmune binding assay (RBA). The difference between ELISA and RBA is that the former can detect varying affinities of IA, while the latter primarily measures high-affinity IA [29]. In the present study, we used RBA to measure the IA titer of patients and demonstrated that IA, even those with the high-affinity property, have a significant effect on the GV of diabetic patients.

CGM is an advanced and useful tool to evaluate overall blood glucose profiles, and it thus provides a unique opportunity to determine the GV of diabetic patients [30]. Using CGM data, clinical researchers and clinicians can effectively assess the quality of the glycemic control of different therapy regimens by calculating the different parameters of GV. MAGE, which was designed to express the glycemic peaks and nadirs, has already become the most frequently used measurement of GV in statistics. An investigation by Su et al. [31] shows that GV is associated with the severity of coronary artery disease (CAD), expressed as the Gensini score, in newly diagnosed T2DM patients. Further analysis indicates that MAGE ≥ 3.4 mmol/l is an independent predictor of CAD in those populations and the predictive value of MAGE is higher than that of HbA1c. In addition, based on the CGM data of 434 healthy subjects, Zhou and his coworkers [32] recommended 3.9 mmol/l as the upper limit of the normal reference range for MAGE in the Chinese adults. In the present study, the ratios of MAGE < 3.9 mmol/l were 48.9%, 24.0%, and 18.0% in the C, L, and H groups, respectively, indicating that the majority of IA-positive patients should change their current therapy regimen to decrease their risk of developing CAD.

The main difference between our study and previous clinical studies [23–26] was that we recruited IA-positive T2DM patients, not newly diagnosed and drug-naïve T2DM patients, as the object of the study. Partially consistent with previous studies [23–26], the HbA1c was positively correlated with MAGE, while the BMI, C-p 0′, and C-p 120′ were negatively correlated with MAGE. Also, the dominant independent predictor of GV was IA in the present study. IA has a long halftime in circulation. Although its titer gradually decreases within one month, its full disappearance requires more than one year after the withdrawal of insulin therapy [33]. Furthermore, we provide evidence that patients with low-titer positive IA (5-15.53%) exhibit larger MAGE than IA-negative subjects, even at similar HbA1c levels. Thus, patients with a higher IA titer might undergo increased GV for a longer period of time if they continue their therapy regimen.

There were limitations in the current study. Firstly, intact 24 h CGM data were only available for enrolled patients who maintained their previous therapy regimen after admission in our medical records. Secondly, we could not calculate the indexes of insulin sensitivity or beta cell function and put them into the regression analysis. This was because the existence of IA hindered the accurate measurement of the fasting or postprandial insulin levels of patients.

## 5. Conclusions

A higher circulating IA titer was associated with increased GV in T2DM patients, indicating that patients with elevated IA titer while being treated with recombinant human insulin or human insulin analogues should undergo CGM for a GV assessment. If GV is undesirable, changing insulin formulations [34], withdrawal of insulin therapy, and/or switching to novel antidiabetic agents are all individualized and appropriate ways to minimize the GV.

## Figures and Tables

**Table 1:** Baseline characteristics of patients.

	C (IA ≤ 5)	L (IA 5-15.53)	H (IA > 15.53)	*F* value/chi-square	*p* value
Age (year)	62.87 ± 10.58	63.64 ± 11.94	62.16 ± 10.27	0.228	0.797
Gender (M/F)	25/22	22/28	23/27	0.900^d^	0.638
Duration (month)	132 (120, 240)	174 (120, 240)	120 (72, 195)	5.134^c^	0.077
BMI (kg/m^2^)	24.90 ± 3.15	24.63 ± 3.66	24.63 ± 2.76	0.118	0.888
IA (%)	2.97 (1.76, 4.15)	8.80 (6.48, 11.76)	31.72 (21.80, 43.72)		
Ln(IA) (%)	0.94 ± 0.58	2.16 ± 0.32^a^	3.42 ± 0.40^ab^	376.120	<0.001
HbA1c (%)	8.54 ± 1.62	8.43 ± 1.43	8.55 ± 1.48	0.095	0.910
Sqrt (C-p 0′) (ng/ml)	1.18 ± 0.46	1.11 ± 0.36	1.17 ± 0.42	0.532	0.588
Sqrt (C-p 120′) (ng/ml)	1.74 ± 0.56	1.73 ± 0.57	1.67 ± 0.65	0.181	0.835
C-p 0′ (-/+)	23/24	17/33	18/32	2.641	0.267
C-p 120′ (-/+)	8/39	7/43	6/44	0.504	0.777
Insulin dose 1 (U/kg)	0.30 (0.24, 0.37)	0.30 (0.23, 0.40)	0.28 (0.20, 0.36)	2.619	0.270
Insulin dose 2 (U/kg)	0.20 (0.13, 0.28)	0.22 (0.18, 0.33)	0.19 (0.16, 0.26)	3.864	0.145
Daily insulin dose (U/kg)	0.51 (0.40, 0.60)	0.53 (0.42, 0.73)	0.49 (0.35, 0.59)	2.850	0.241
Insulin/insulin analogue	14/33	12/38	13/37	0.427^d^	0.808
Oral drugs					
Met (-/+)	41/6	45/5	40/10	2.172^d^	0.338
Aca (-/+)	38/9	40/10	42/8	0.295^d^	0.863
Met+Aca (-/+)	43/4	48/2	44/6	2.218^d^	0.337

BMI: body mass index; IA: insulin antibody; Ln: base *e* logarithm; HbA1c: glycosylated hemoglobin A1c; Sqrt: square root; C-p: C-peptide; C-p 0′ (-): C-p 0′ ≤ 1.10 ng/ml; C-p 0′ (+): C-p 0′ > 1.10 ng/ml; C-p 120′ (-): C-p 120′ ≤ 1.10 ng/ml; C-p 120′ (+): C-p 120′ > 1.10 ng/ml; insulin dose 1: insulin dose before breakfast; insulin dose 2: insulin dose before dinner; Met: metformin; Aca: acarbose. ^a^Compared with the C group (*p* < 0.05); ^b^compared with the L group (*p* < 0.05); ^c^nonparametric test; ^d^Chi-square test. Data were presented as mean ± SD or median (25th, 75th percentile).

**Table 2:** Glycemic variability among different IA titer groups.

	C	L	H	*F* value/chi-square	*p* value
Ln(MAGE) (mmol/l)	1.36 ± 0.37	1.60 ± 0.35^a^	1.79 ± 0.50^ab^	12.610	<0.001
Ln(SDBG) (mmol/l)	0.55 ± 0.37	0.73 ± 0.35^a^	0.84 ± 0.44^a^	6.984	0.001
LAGE (mmol/l)	7.49 ± 2.39	9.15 ± 2.72^a^	10.22 ± 3.75^a^	9.928	<0.001
MBG (mmol/l)	9.41 ± 2.05	9.49 ± 1.96	9.64 ± 1.82	0.170	0.844
AUC > 13.9 (mmol/l∗min)	0.00 (0.00, 98.25)	7.55 (0.00, 283.48)	173.79 (0.00, 565.20)^ab^	10.492^c^	0.005
PT1 (%)	0.00 (0.00, 10.07)	1.39 (0.00, 13.89)	9.10 (0.00, 22.31)^ab^	8.100^c^	0.017
AUC < 3.9 (mmol/l∗min)	0.00 (0.00, 0.00)	0.00 (0.00, 2.80)	0.00 (0.00, 17.92)^a^	6.088^c^	0.048
PT2 (%)	0.00 (0.00, 0.00)	0.00 (0.00, 1.31)	0.00 (0.00, 2.53)^a^	5.391^c^	0.048

Ln: base *e* logarithm; MAGE: the 24 h mean amplitude of glycemic excursion; SDBG: the standard deviation of 24 h blood glucose; LAGE: the large amplitude of glycemic excursion; MBG: the 24 h mean blood glucose concentration; AUC > 13.9 mmol/l: the incremental area under the curve of plasma glucose > 13.9 mmol/l; AUC < 3.9 mmol/l: the incremental area under the curve of plasma glucose < 3.9 mmol/l; PT1: the percentage of the time spent on glucose concentrations above 13.9 mmol/l; PT2: the percentage of the time spent on glucose concentrations below 3.9 mmol/l. ^a^Compared with the C group (*p* < 0.05); ^b^compared with the L group (*p* < 0.05); ^c^nonparametric test. Data were presented as mean ± SD or median (25th, 75th percentile).

**Table 3:** The correlation between Ln(MAGE) and different parameters.

Parameter	*r*	*p* value
Age	0.098	0.239
Gender	−0.098	0.238
Duration	−0.054	0.517
Sqrt (C-p 0′)	−0.180	0.030
Sqrt (C-p 120′)	−0.229	0.005
BMI	−0.263	0.001
HbA1c	0.203	0.014
Ln(IA)	0.404	<0.001
Ln(IA)^∗^	0.423	<0.001

Sqrt: square root; C-p: C-peptide; Ln: base *e* logarithm; IA: insulin antibody; BMI: body mass index; HbA1c: glycosylated hemoglobin A1c. ^∗^After adjusting for age, gender, BMI, duration, Sqrt (C-p 0′), Sqrt (C-p 120′), and HbA1c.

**Table 4:** Stepwise multiple linear regression analysis with Ln(MAGE) as the dependent variable.

	Adjusted *R*^2^	Unstandardized *β*	*β* Std. error	Standardized *β*	*t*	*p* value
Model 1	0.158					<0.001
Constant		1.226	0.076		16.103	<0.001
Ln(IA)		0.163	0.031	0.405	5.271	<0.001
Model 2	0.210					<0.001
Constant		2.070	0.273		7.588	<0.001
Ln(IA)		0.158	0.030	0.391	5.258	<0.001
BMI		−0.034	0.010	−0.239	−3.211	0.002
Model 3	0.239					<0.001
Constant		1.515	0.346		4.378	<0.001
Ln(IA)		0.159	0.029	0.395	5.399	<0.001
BMI		-0.030	0.010	−0.215	−2.921	0.004
HbA1c		0.055	0.022	0.186	2.528	0.013

Ln: base *e* logarithm; IA: insulin antibody; BMI: body mass index; HbA1c: glycosylated hemoglobin A1c.

## Data Availability

The data sets generated during and/or analyzed during the current study are not publicly available but are available from the corresponding author on reasonable request.
